# Sigma metric revisited: True known mistakes

**DOI:** 10.11613/BM.2019.010902

**Published:** 2018-12-15

**Authors:** Abdurrahman Coskun, Mustafa Serteser, Ibrahim Ünsal

**Affiliations:** Department of Medical Biochemistry, Acıbadem Mehmet Ali Aydınlar University, School of Medicine, Istanbul, Turkey

**Keywords:** laboratory error, normal distribution, sigma metric, Six Sigma, uniform distribution

## Abstract

Six Sigma methodology has been used successfully in industry since the mid-1980s. Unfortunately, the same success has not been achieved in laboratory medicine. In this case, although the multidisciplinary structure of laboratory medicine is an important factor, the concept and statistical principles of Six Sigma have not been transferred correctly from industry to laboratory medicine. Furthermore, the performance of instruments and methods used in laboratory medicine is calculated by a modified equation that produces a value lower than the actual level. This causes unnecessary, increasing pressure on manufacturers in the market. We concluded that accurate implementation of the sigma metric in laboratory medicine is essential to protect both manufacturers by calculating the actual performance level of instruments, and patients by calculating the actual error rates.

## Introduction

Six Sigma methodology is the latest version of total quality management and has been widely used in industry since the mid-1980s. One of the most powerful aspects of Six Sigma is its universal applicability to various fields including business, health care, and laboratory medicine. Unfortunately, despite great success of the Six Sigma methodology in industry, the same success has not been achieved in laboratory medicine.

Quality principles have not been applied to laboratory medicine as rigorously as industry. Interestingly (probably psychologically), the expected success or error rates in industry and laboratory medicine are not the same. For example, in laboratory medicine, 4 Sigma quality is accepted as a success, but in the aviation sector the target is 7 Sigma.

Although the principles of the Six Sigma methodology are universal, calculations of the sigma metric (SM) in industry and laboratory medicine differ. The reasons for the different calculation of SM in laboratory medicine need to be investigated. In our previous studies, we have shown that the conventional equation used to calculate SM in laboratory medicine is different from the equation used in industry (*1*). Additionally, the standard equation for SM has not been transferred correctly from industry to laboratory medicine (*1*-*3*). Therefore, the equation used to calculate SM in laboratory medicine should be critiqued and corrected.

In this paper, we aimed 1) to explain the statistical techniques of how engineers calculate short and long-term SM and error rates in industry and business, and 2) to show the defects of the SM equation used in laboratory medicine.

## Sigma metric in industry and business

In industry, SM is calculated as given below (Equation (Eq.) 1):
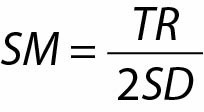
where TR is the tolerance range of the product, and SD is the standard deviation of the process. Tolerance range can be calculated from the subtraction of lower tolerance limit (LTL) from the upper tolerance limit (UTL) (UTL – LTL). At the 6 Sigma quality level, 12 SD fit between the lower and upper TLs, *i.e*., 6 SD fits between the target and LTLs/UTLs.

The SM value indicates the performance of the process; the lower the SM value the process has, the higher error rate the process produces. To illustrate further, we explain the meaning of SM in terms of errors. For this purpose, we use defects *per* million opportunities (DPMO), which is the number of defective products within 1 million opportunities. For example, if DPMO is 1000, it means that within 1 million opportunities 1000 products are defective, and the rest are acceptable. The conversion of SM to DPMO and *vice versa* makes Six Sigma methodology a powerful tool in both industry and business. It serves as a bridge between counting and variation methodology and therefore it is accepted as a universal tool to measure the performance of processes in both industry and business. In laboratory medicine, only variation or counting variables cannot be used to evaluate the performance of the whole laboratory. For example, in analytical phase the main variable is SD but in pre-pre-analytical phase it is DPMO. Therefore, in a Six Sigma project of total testing process, we have to convert all data to DPMO and then focus on the phase which is the center of errors/defects.

The SMs and corresponding DPMOs are given in Table 1. As shown in Table 1, for each SM value there are 2 different DPMOs, short and long terms (*4*). In daily practice we prefer the long-term DPMO (*4*). It has been questioned why we prefer long-term SM. No system is perfect and every process deviates from its target in time. The Six Sigma methodology was developed and applied to processes by William B. Smith and Motorola Inc. engineers. During the early period of observations, engineers found that processes deviated approximately 1.5 SD from the targets. In a given time interval, we do not know the exact shift (bias) from the target. Acceptance of a 1.5 SD shift from the target is a form of insurance in estimating the quality of the process. Therefore, engineers in industry usually include the 1.5 SD shift from the target and calculate the DPMO corresponding to the SM accordingly.

**Table 1:** Defects *per* million opportunities corresponding to long and short term sigma metrics

**SM**		**Long Term**				**Short Term**		
	**Z_LTL_ (AUC)**	**Z_UTL_ (AUC)**	**Z_UTL-LTL_ (AUC)**	**DPMO_L_**	**Z_LTL_ (AUC)**	**Z_UTL_ (AUC)**	**Z_UTL-LTL_ (AUC)**	**DPMO_S_**
**1.0**	(0.493790335)	(- 0.191462461)*	(0.3023278735)	697,670	(0.3413447458)	(0.3413447458)	(0.6826894917)	317,310
**1.5**	0.498650102	0.0000000000	0.498650102	501,350	0.4331927985	0.4331927985	0.8663855971	133,610
**2.0**	0.499767371	0.191462461	0.6912298320	308,770	0.4772498679	0.4772498679	0.9544997359	45,500
**2.5**	0.499968329	0.341344746	0.8413130746	158,690	0.4937903346	0.4937903346	0.9875806693	12,420
**3.0**	0.499996602	0.433192799	0.9331894009	66,810	0.498650102	0.498650102	0.9973002039	2700
**3.5**	0.499999713	0.477249868	0.9772495813	22,750	0.499767371	0.499767371	0.499767371	465
**4.0**	0.499999981	0.493790335	0.9937903156	6210	0.499968329	0.499968329	0.9999366575	63
**4.5**	0.499999999	0.498650102	0.9986501010	1350	0.499996602	0.499996602	0.9999932047	6.8
**5.0**	0.4999999999	0.499767371	0.9997673709	233	0.499999713	0.499999713	0.9999994267	0.57
**5.5**	0.4999999999	0.499968329	0.9999683278	32	0.499999981	0.499999981	0.9999999620	0.04
**6.0**	0.4999999999	0.499996602	0.9999966013	3.4	0.499999999	0.499999999	0.9999999980	0.002
We use z score and z table to calculate the DPMOs corresponding to SMs. Area under the curve (AUC) obtained from z table. *Due to 1.5 SD shift in long term SM, the Z_UTL_ is higher than UTL and therefore the AUC of Z_UTL_ was subtracted from the AUC of Z_LTL_. SM – Sigma metric. LTL - lower tolerance limit. UTL – upper tolerance limit. DPMO – defects *per* million opportunities.								

Next, we examine how engineers calculate the DPMO corresponding to short and long-term SM. The calculation of DPMO (both short and long term) is based on the normal distribution curve. The equation of the normal distribution function is given below (Eq. 2).
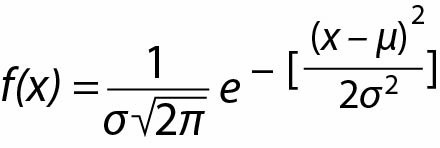
where f(x) is the normal distribution function, σ is the standard deviation, μ is the mean, and x is a variable.

The DPMO corresponding to SM is derived from the area under the curve (AUC) restricted by LTL and UTL as calculated below (Eq. 3):


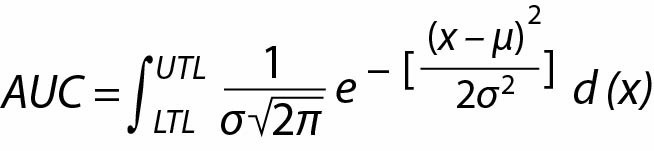


This equation gives AUC but is rather complex to be used in daily practice. For simplicity, we use the standard normal distribution curve. In this curve, the target is 0 and SD is 1.

To calculate the DPMO corresponding to SM, in the first step, we calculate the AUC restricted by LTL and UTL. The total AUC (-∞ to +∞) is 1. The error rate is 1-AUC and therefore the DPMO can be calculated as given below (Eq. 4):


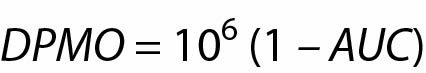


Although this equation is very simple, we still must calculate the restricted AUC, which is not easy. To overcome this problem, we use tables instead of complex equations. For the standard normal distribution curve, we use the z table.

To calculate the DPMO corresponding to SM, we find the AUC from the LTL to UTL from the z table and use Eq. 4. For example, to calculate the DPMO corresponding to 5 SM, we find the AUC restricted by - 5 to 5 from z table and then use Eq. 4 as given below:

AUC from − 5 to 0 = 0.4999997133

AUC from 0 to 5 = 0.4999997133

AUC from − 5 to 5 = 0.9999994266

DPMO = 10^6^ (1 − 0.9999994266) = 0.57.

**Figure 1:**
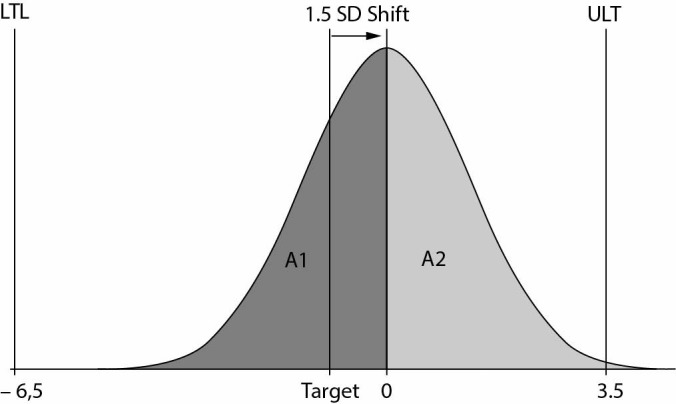
To calculate the long-term DPMO of 5 SM we find the area under the curve from − 6.5 to 3.5. Due to the 1.5 SD shift, the limit of the left tail of the curve is − 6.5 (− 1.5 - 5) and the limit of the right tail of the curve is 3.5 (5 - 1.5). The DPMO corresponding to SMs are calculated using Eq. 4.

This is the short-term DPMO, but in daily practice we use the long-term SM and corresponding DPMO. To calculate the long term DPMO we include the shift in the calculations. In this case we find the AUC from (- shift - LTL) to (UTL + Shift) from the z table. For example, if we want to calculate the long-term DPMO of 5 SM we find the AUC from − 6.5 to 3.5. Due to the 1.5 SD shift, the limit of the left tail of the curve is − 6.5 (−1.5 - 5) and the limit of the right tail of the curve is 3.5 (5 -1.5) (Figure 1).

The DPMO corresponding to SMs are calculated using Eq. 4 as given below:

AUC from − 6.5 to 0 = 0.4999999999

AUC from 0 to 3.5 = 0.4997673709

AUC from − 6.5 to 3.5 = 0.9997673708

DPMO = 10^6^ (1 - 0.9997673708) = 233.

From these simple examples we can see that both short and long term DPMOs are calculated using the standard normal distribution curve (Table 1). However, in daily practice it is not necessary to use complex mathematical equations. We can use Eq. 1 to calculate SM and then Table 1 to find the corresponding DPMO. Engineers in industry use Eq. 1 and Table 1 to calculate SM and DPMO easily.

## Sigma metric in laboratory medicine

In laboratory medicine literature, the scientific background of Six Sigma methodology is not well understood. In our previous studies we aimed to implement the corrected SM in laboratory medicine and calculate the performance of the instruments accurately, but it seems that there are still misunderstandings in the field (*1*-*3*). For example, recently Bayat criticized our paper and stated that *“Coskun et al. confused the short-term/long-term concept with the one-sided/two-sided concept. The reason that the calculated defect rates in the Coskun et al. calculations are significantly lower than the Westgard approach is that they have neglected the 1.5 SD subtraction.”* (*1*, *5*). We believe that our paper is a seminal paper and had not been read in detail by Bayat (*1*). In the paper, we criticized all aspects of the defects of the SM calculation method in laboratory medicine. Additionally, we never neglected the 1.5 SD subtraction, but treated it correctly.

In laboratory medicine, a different equation is used to calculate SM as given below (Eq. 5):


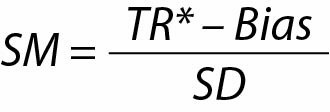


TR* is the range from the center (target) to UTL or LTL. This equation is different from the main SM equation (Eq. I), it includes bias and structurally similar to process capability index (Cpk) as given below (Eq. 6):
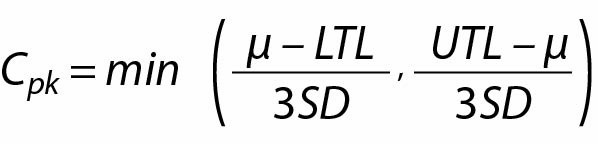
where µ is the mean of the process.

It should be noted that SM and Cpk are not the same. Sigma metric is linked to DPMO and must be interpreted correctly. The difference between SM and Cpk is the subject of another paper and cannot be summarized in this short paper.

There are two major drawbacks of calculating SM from Eq. 5.

**Figure 2:**
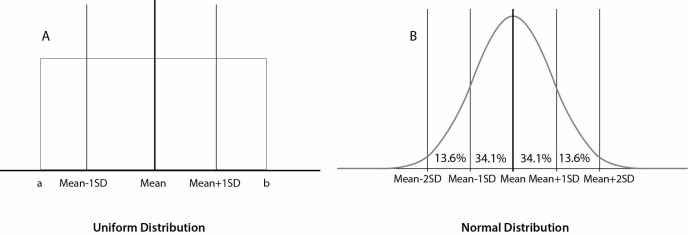
A linear relation is present in uniform distributions (A), but not in normal distributions (B). Moving the mean to the right or left increases or decreases the AUC linearly in the uniform distribution, but not in the normal distribution. Therefore, inclusion of bias as a linear component in Eq. 5 is mathematically not valid. The SD of uniform distribution is [(b-a)/12]^1/2^.

1. Mathematical defects and illogical results. We derive SM and DPMO from the normal distribution curve but the relation between these parameters is not linear. A linear relation is present in uniform distributions, not in normal distributions (Figure 2). As shown in Figure 2, moving the mean to the right or left increases or decreases the AUC linearly in the uniform distribution but not in the normal distribution. Inclusion of bias as a linear component in Eq. 5 is mathematically not valid. In statistics there are various distributions types and for SM we must follow the mathematics of normal distribution (*6*).

**Figure 3:**
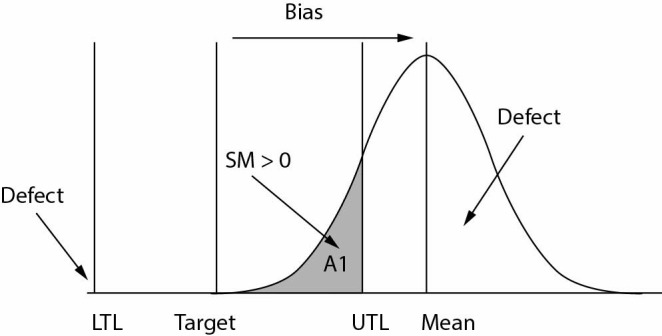
A normal distribution curve is a two-sided curve (from −∞ to +∞) and the tails of the curve do not intersect the x-axis. Bias might be on the right or left side of the mean. Even if the bias is larger than UTL or LTL, the performance of a working process is always higher than zero. Normal distribution curve is the mathematical reference of both SM and DPMO. Negative SM cannot be obtained from normal distribution curve.

Equation 5 is a one-sided and linear treatment of bias open to illogical results such as negative Sigma. If the bias is larger than UTL/LTL, according to Eq. 5, SM will be a negative value (Figure 3). Negative performance is meaningless, in both theory and practice. The lowest level of the performance of a process is zero, not a negative value. A negative SM would mean that the process produces more errors than there are opportunities. For example, if we have 1 million products the maximum error rate is 1 million (all products are defective). It is not logical to state that more than 1 million products of 1 million products are defective.

Mathematically we can show that the performance of a working process is always > 0 (Figure 3). The performance of the process is derived from the AUC restricted by LTL and UTL. The normal distribution curve is a two-sided curve (from −∞ to +∞) and the tails of the curve do not intersect the x-axis and therefore the AUC is always > 0.

In industry for a given SM value, we have one short (no shift) and one long term (shift is 1.5 SD) DPMO. But according to Eq. 5, for a given SM value we have one short (no shift) but can obtain a lot of long term DPMOs. For example, if SM = 1, the short-term DPMO will be 317,310 (Table 1), but the long-term SM may correspond to various DPMOs such as 697,670 (Table 1), 158,650, 317,310 (Table 2) or even a different value. This is because Eq. 5 has two variables: bias and CV. If we change the values of bias and CV, we can obtain different DPMOs correspond the same SM (Table 2). This is a clear contradiction. In mathematics and philosophy this is a typical example of *reductio ad absurdum.* The inclusion of bias as a linear component cause absurd results.

**Table 2:** Linear treatment of bias in standard normal distribution curve creates nonsense results

**LTL**	**UTL**	**Target**	**Bias**	**CV**	**SM**	**Z_LTL_ (AUC)**	**Z_UTL_ (AUC)**	**Z_LTL-UTL_ (AUC)**	**DPMO**
**Urea nitrogen, mg/dL**									
**16**	24	20	3.0	1.0	**1**	- 7.0 (0.4999999999)	1 (0.3413447458)	- 7 to + 1 (0.8413447458)	158,650*
**16**	24	20	2.0	2.0	**1**	- 3.0 (0.4986501019)	1 (0.3413447458)	- 3 to + 1 (0.8399948477)	**160,000**
**16**	24	20	0.0	4.0	**1**	- 1.0 (0.3413447458)	1 (0.3413447458)	- 1 to + 1 (0.6826894917)	317,310**
**Low-density lipoprotein cholesterol, mg/dL**									
**88**	112	100	3.0	3.0	**3**	- 5.0 (0.4999997133)	3 (0.4986501020)	- 5 to + 3 (0.9986498153)	1350*
**88**	112	100	1.5	3.5	**3**	- 3.9 (0.4999433065)	3 (0.4986501020)	- 3.9 to + 3 (0.9985934084)	**1410**
**88**	112	100	0.0	4.0	**3**	- 3.0 (0.4986501020)	3 (0.4986501020)	- 3 to + 3 (0.9973002039)	2700**
For the same SM, we can obtain various DPMOs. In this table for simplicity we show only three different DPMOs corresponding to the same SM. For the same test, the DPMO of (**) is approximately 2 times higher than the DPMO of (*). LTL - lower tolerance limit. UTL – upper tolerance limit. CV – coefficient of variation. SM – Sigma metric. DPMO – defects *per* million opportunities. AUC - Area under the curve									

2. Application defect. If we include bias directly, we must use the short-term DPMO, not the long-term DPMO. For example, if the TR, Bias, and SD of a process are 12, 4, and 2 respectively, then according to Eq. 5 (although mathematically it is not correct) the SM will be 4. Now the question is what is the DPMO corresponding to 4 Sigma. The answer is 63, not 6200 (Table 1). Because we know the shift of the process, we must use the DPMO corresponding to the short-term SM. Unfortunately, in various papers (and in laboratory practice), bias is directly included in the equation of SM, and the DPMO corresponding to the long-term SM is used. If we use Eq. 5 in the performance calculations of analysers, methods, reagents, and other instruments, we will obtain performance values significantly lower than the actual levels. This results from two biases: the bias directly included in the equation, and the 1.5 SD shift accepted as the natural bias. This false low performance level will increase pressure on the manufacturers in the market. Additionally, the fact that one SM corresponds to various DPMO makes the application of Eq. 5 meaningless in laboratory medicine.

## Conclusion

As mentioned previously, engineers in industry use Eq. 1, in which bias is not directly included, because mathematically it is not valid. Instead of directly including bias, they prefer to use the long-term DPMO corresponding to SM. This approach has two important advantages: first, the equation is very simple and it is easy to calculate the performance of a process using the TR and SD. Second, in daily practice we do not know whether bias exists or not, and therefore we accept the presence of a 1.5 SD shift in any case. This assumption protects the process performance evaluation from the possible presence of biases.

The approach used in laboratory medicine has serious defects. First, it includes bias measured in laboratory and uses DPMO corresponding to long term SM. It should be noted that, in laboratory medicine, the bias measurement methods differ among laboratories and some of them are defective, thus the results may not be reliable. The SM calculated using Eq. 5 will be very low; consequently the DPMO will be very high. This results from two biases: the measured bias, and the 1.5 SD shift. In this case, the calculated performance of the process will be incorrect, significantly lower than the actual performance level, causing serious trouble for manufacturers in the market. To overcome this problem we have two choices. First, we can perform the calculations as engineers do in industry, by only using TR and SD to calculate SM, and then use the long-term DPMO. Second, if we include bias we should use z score and z table to calculate short term DPMO.
